# Effects of Bisphenol A Metabolite 4-Methyl-2,4-bis(4-hydroxyphenyl)pent-1-ene on Lung Function and Type 2 Pulmonary Alveolar Epithelial Cell Growth

**DOI:** 10.1038/srep39254

**Published:** 2016-12-16

**Authors:** Shing-Hwa Liu, Chin-Chuan Su, Kuan-I Lee, Ya-Wen Chen

**Affiliations:** 1 Institute of Toxicology, College of Medicine, National Taiwan University, Taipei, Taiwan; 2 Department of Medical Research, China Medical University Hospital, China Medical University, Taichung, Taiwan; 3 Department of Otorhinolaryngology, Head and Neck Surgery, Changhua Christian Hospital, Changhua County, Taiwan; 4 Department of Emergency, Taichung Tzuchi Hospital, Taichung, Taiwan; 5 Department of Physiology and Graduate Institute of Basic Medical Science, College of Medicine, China Medical University, Taichung, Taiwan

## Abstract

Bisphenol A (BPA) is recognized as a major pollutant worldwide. 4-Methyl-2,4-bis(4-hydroxyphenyl)pent-1-ene (MBP) is a major active metabolite of BPA. The epidemiological and animal studies have reported that BPA is harmful to lung function. The role of MBP in lung dysfunction after BPA exposure still remains unclear. This study investigated whether MBP would induce lung alveolar cell damage and evaluated the role of MBP in the BPA exposure-induced lung dysfunction. An *in vitro* type 2 alveolar epithelial cell (L2) model and an *ex vivo* isolated reperfused rat lung model were used to determine the effects of BPA or MBP on cell growth and lung function. MBP, but not BPA, dose-dependently increased the mean artery pressure (Pa), pulmonary capillary pressure (Pc), pulmonary capillary filtration coefficient (K_fc_), and wet/dry weight ratio in isolated reperfused rat lungs. MBP significantly reduced cell viability and induced caspases-3/7 cleavage and apoptosis and increased AMP-activated protein kinas (AMPK) phosphorylation and endoplasmic reticulum (ER) stress-related molecules expression in L2 cells, which could be reversed by AMPK-siRNA transfection. These findings demonstrated for the first time that MBP exposure induced type 2 alveolar cell apoptosis and lung dysfunction through an AMPK-regulated ER stress signaling pathway.

The worldwide production of bisphenol A (BPA) is approximately 3.2 million tons per year^1^. The BPA is a well-known chemical and widely applied in the manufacture of polycarbonate plastic containers^2^. Human exposure to BPA is widespread. A study showed that oral consumption of canned soup might cause a short-term 1000-fold increase in plasma BPA concentration^3^. 4-Methyl-2,4-bis(4-hydroxyphenyl)pent-1-ene (MBP) has been demonstrated to be an active metabolite of BPA by NMR and LC/MS/MS analysis^4^. The MBP accumulation in human might be due to oral, dermal, and inhalation after BPA exposure and ingestion^5^,^6^. It has also been found that BPA can release into the aquatic environment and converted to MBP, which results about 250-fold toxicity than BPA on the medaka (*Oryzias latipes*)^7^. Yoshihara *et al*. have also shown that MBP possesses more potent estrogenic activity than BPA in several *in vitro* tests^4^.

Several studies reported that BPA was harmful to lung function^3^,^8^,^9^. A study investigated the effects of BPA on lung function in 208 children. It showed that prenatal BPA exposure during early gestation increased the risk of wheeze and led to a persistent wheeze phenotype, which was associated with a change in forced expiratory volume in 1 s (FEV_1_; an FEV_1_/forced vital capacity (FVC) ratio of ≤ 80% is suggested to indicate obstructive lung disease)^9^. BPA has been shown to increase the production of the pro-inflammatory cytokine interleukin-4 in helper T cells and the levels of antigen-specific immunoglobulin E, which is associated with allergic immune responses^10^. Another study also showed that maternal BPA exposure increased the numbers of eosinophils in the bronchoalveolar lavage fluid and airway hyper-responsiveness in mouse pups^3^,^8^.

There are two types of alveolar epithelial cells in the lung: alveolar type 1 and alveolar type 2 cells. Type 2 alveolar epithelial cells produce and secrete surfactant to reduce the surface tension and maintain the patency of the alveoli and distal airway. Moreover, type 2 alveolar epithelial cells can serve as stem cells to restore and turn over alveolar epithelial cells during lung injury and development^11^,^12^. Many studies have shown that type 2 alveolar epithelial cell impairment leads to the development of lung diseases, including chronic obstructive pulmonary disease (COPD), acute respiratory distress syndrome (*ARDS*), and lung fibrosis^13^,^14^,^15^,^16^.

There were no studies that investigated whether BPA metabolite MBP plays a role in causing lung dysfunction after BPA exposure. Hence, we hypothesized that MBP may contribute to the deterioration in pulmonary alveolar epithelial cell growth and lung function following BPA exposure. To demonstrate this hypothesis, we used an *in vitro* type 2 alveolar epithelial cell model and an *ex vivo* isolated reperfused rat lung model to investigate the effects of MBP on type 2 pulmonary alveolar epithelial cell growth and lung function.

## Results

### Both BPA and its metabolite MBP reduced cell viability in L2 alveolar epithelial cells

**Figure 1:**
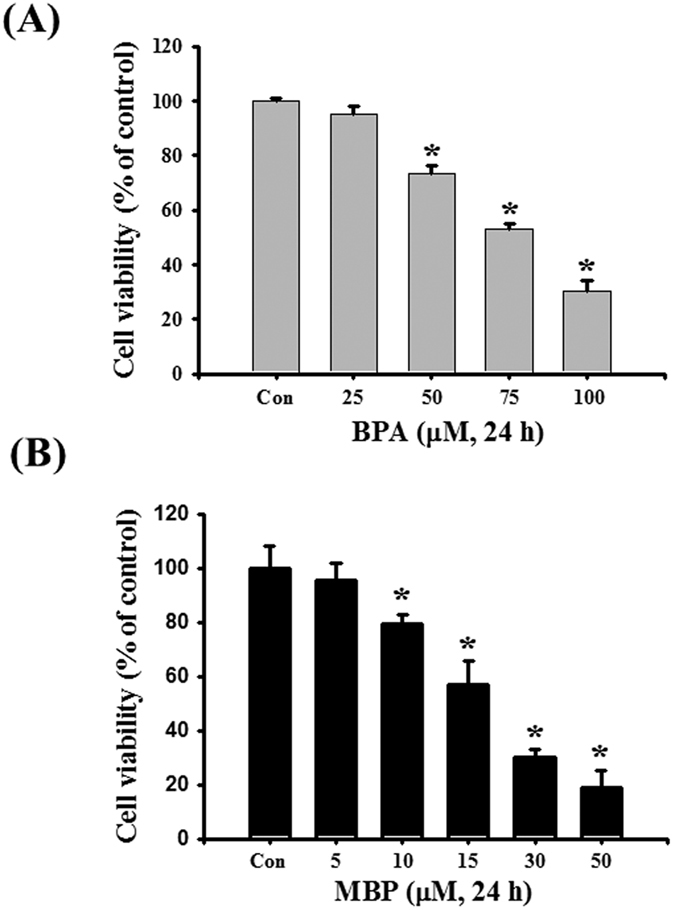
BPA and its metabolite MBP suppressed cell viability in L2 alveolar epithelial cells. Cells were treated with BPA (25–100 μM, (**A**)) or MBP (5–50 μM, (**B**)) for 24 h. Cell viability was determined by MTT assay. Data are presented as mean ± SEM of three independent experiments. *P < 0.05 as compared with the vehicle control group. Con: control.

Treatment with both BPA (25–100 μM) and MBP (5–50 μM) to L2 cells for 24 h significantly and dose-dependently reduced cell viability (Fig. 1A and B and Fig. S1-A and S1-B). The IC50 was about 75 μM for BPA treatment and about 15 μM for MBP treatment.

We next used the IC50 concentrations of BPA and MBP to investigate their effects on isolated reperfused lung model or the possible mechanisms on lung cells damage.

### BPA and MBP induced lung dysfunction in isolated reperfused rat lungs

**Figure 2:**
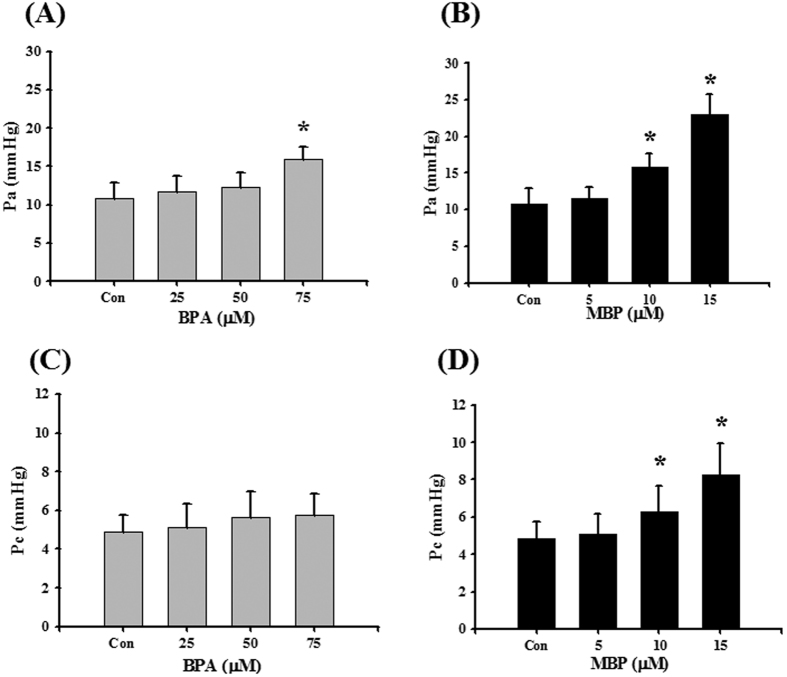
Effects of BPA and its metabolite MBP on pulmonary artery pressure (P_a_) and pulmonary capillary pressure (P_c_) in isolated rat reperfused lung model. Isolated lungs were reperfused with BPA (**A,C**) or MBP (**B,D**) for 120 min. The P_a_ (**A,B**) and P_c_ (**C,D**) were measured by pressure transducers. Data are presented as the mean ± SEM (n ≥ 10). *P < 0.05 as compared with the vehicle control group. Con: control.

**Figure 3:**
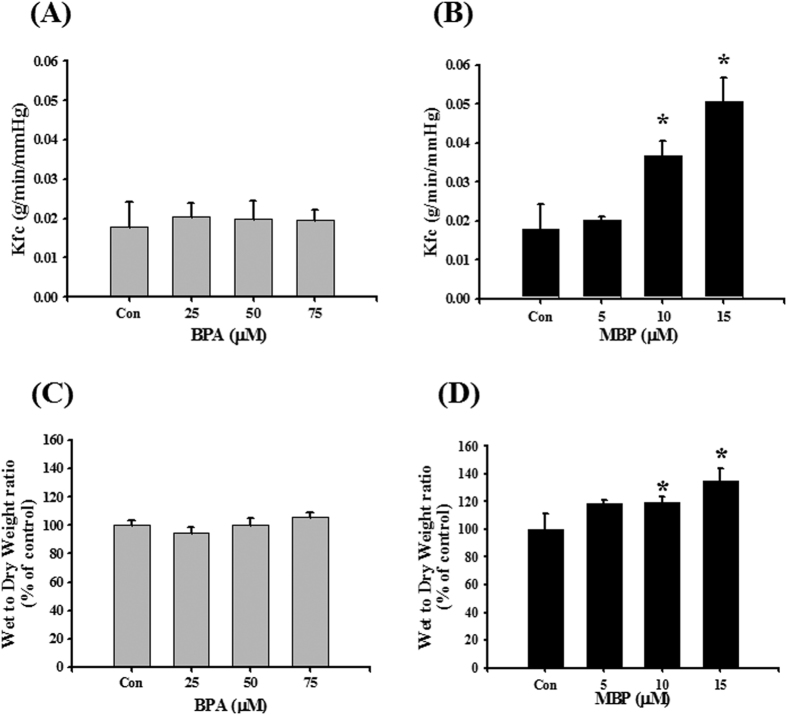
Effects of BPA and its metabolite MBP on pulmonary capillary filtration coefficient (K_fc_) and wet-to-dry weight ratio in isolated rat reperfused lung model. Isolated lungs were reperfused with BPA (**A,C**) or MBP (**B,D**) for 120 min. The K_fc_ (**A,B**) was measured by pressure transducers. Wet-to-dry weight ratio (**C,D**) was obtained after lungs were weighted and dried to a constant weight at 60 °C. Data are presented as the mean ± SEM (n ≥ 10). *P < 0.05 as compared with the vehicle control group. Con: control.

To investigate the effect of BPA and MBP on lung function, an isolated reperfused rat lung model was established. BPA (75 μM) significantly increased P_a_, but did not affect P_c_ (Fig. 2A and C), K_fc_, and wet/dry weight ratio (Fig. 3A and C). MBP (10 and 15 μM) significantly increased P_a_, P_c_ (Fig. 2B and D), K_fc_, and wet/dry weight ratio (Fig. 3B and D). These results indicated that MBP was more potent than BPA in affecting lung function in isolated reperfused rat lungs.

### Both BPA and MBP affected cell cycle and induced apoptosis in L2 alveolar epithelial cells

**Figure 4:**
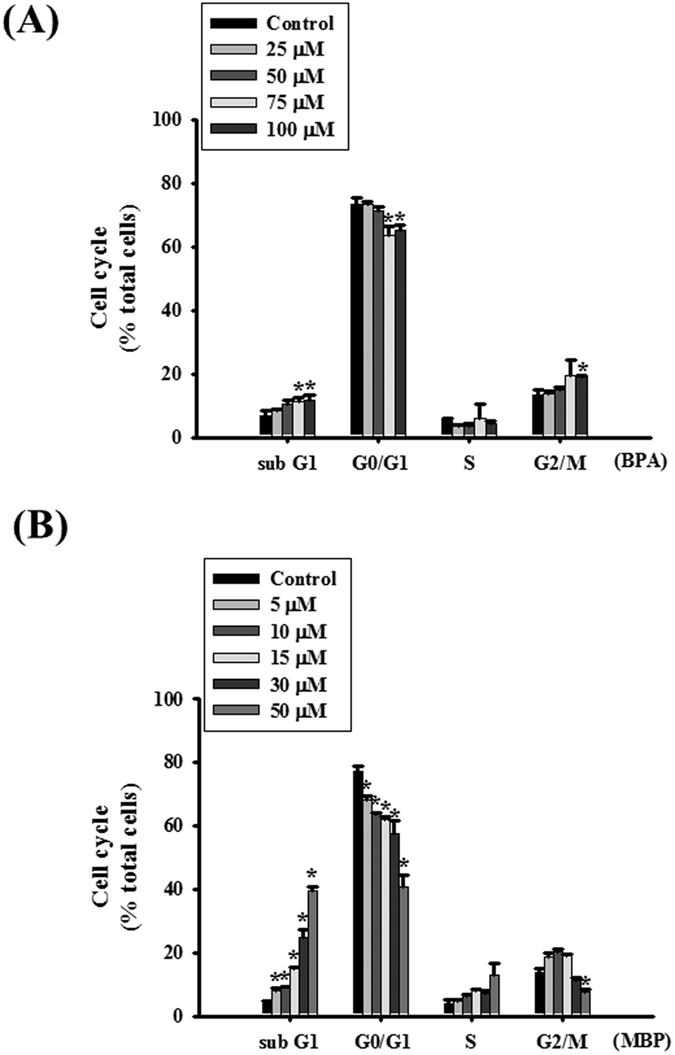
BPA and its metabolite MBP affected cell cycle in L2 alveolar epithelial cells. Cells were treated with BPA (25–100 μM, (**A**)) or MBP (5–50 μM, (**B**)) for 24 h. Cell cycle analysis was determined by flow cytometry. Data are presented as mean ± SEM of three independent experiments. *P < 0.05 as compared with the vehicle control group. Con: control.

As shown in Fig. 4A and B, both BPA (25–100 μM) and MBP (5–50 μM) increased sub-G1 contents and decreased G0/G1 contents in L2 alveolar epithelial cells. MBP was more potent than BPA in affecting cell cycle. These results suggested that MBP was capable of affecting the cell cycle stages.

**Figure 5:**
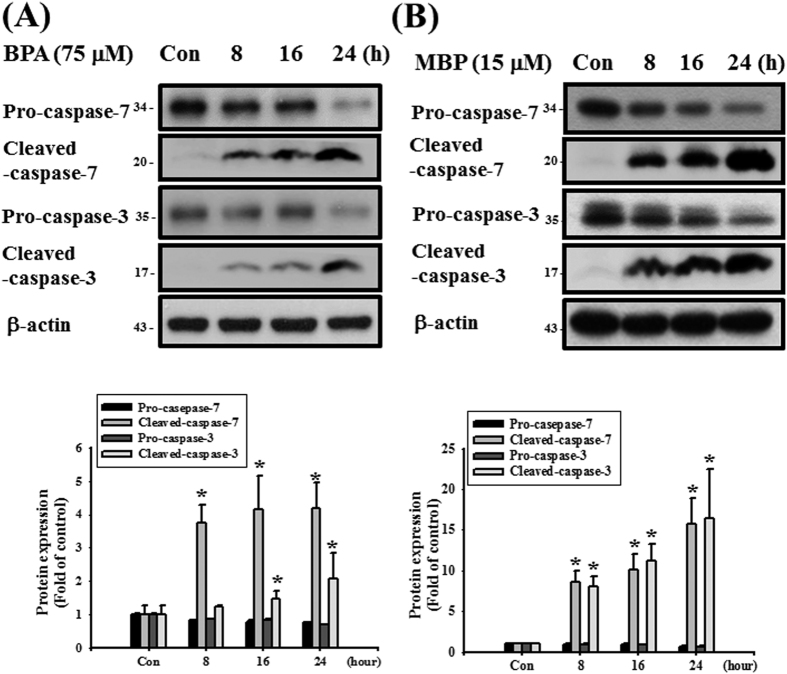
BPA and its metabolite MBP induced apoptotic signals in L2 alveolar epithelial cells. In (**A**,**B**) cells were treated with BPA (75 μM, (**A**)) or MBP (15 μM, (**B)**) for 8, 16, and 24 h. The protein expressions of pro- and cleaved- caspase-7 and caspase-3 proteins were analyzed by Western blotting. Data are representative of three independent experiments. The protein expression of β-actin was as an internal control. The protein expressions were quantified by densitometry and analyzed by ImageQant TL 7.0 software. Data are presented as mean ± SEM of three independent experiments. *P < 0.05 versus vehicle control group.

**Figure 6:**
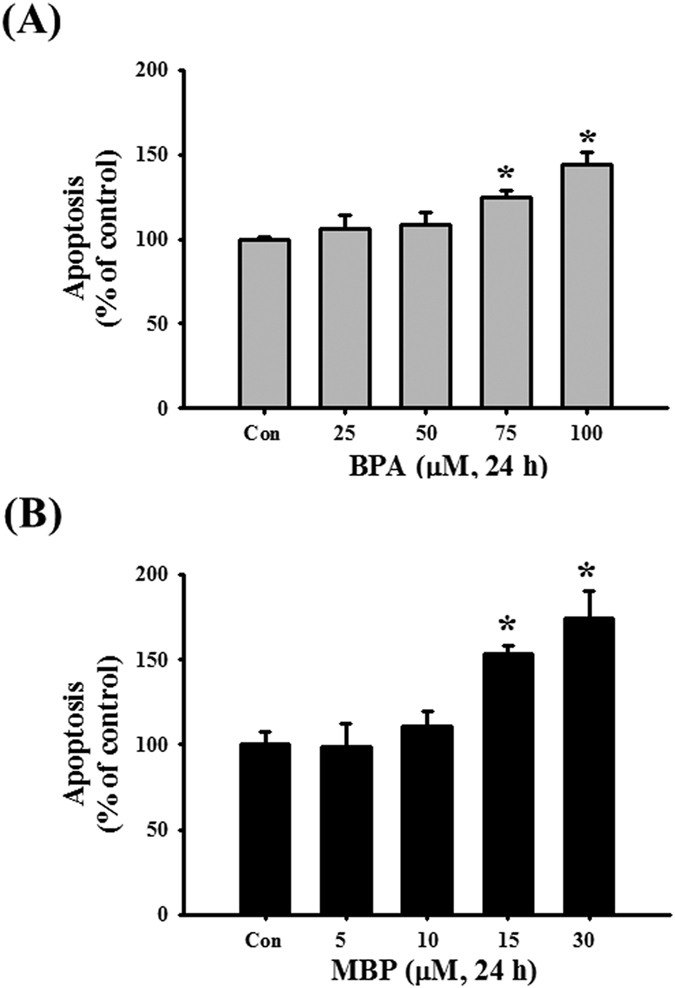
BPA and its metabolite MBP induced apoptosis in L2 alveolar epithelial cells. In (**A,B**) cells were treated with BPA (25–100 μM, (**C**)) or MBP (5–30 μM, (**D**)) for 24 h. The annexin V- FITC staining for apoptosis was determined by flow cytometry. Data are presented as mean ± SEM of three independent experiments. *P < 0.05 as compared with the vehicle control group. Con: control.

We next used the IC50 concentrations of BPA and MBP to investigate whether BPA and MBP would induce cell apoptosis in L2 cells. The results showed that BPA (75 μM) and MBP (15 μM) significantly decreased the levels of pro-caspase-7 and pro-caspase-3 proteins and increased the levels of cleaved caspase-7 and cleaved caspase-3 proteins (Fig. 5A and B). Moreover, the results of annexin-V-FITC staining showed that both BPA (75 and 100 μM) and MBP (15 and 30 μM) treatment significantly increased cell apoptosis (Fig. 6A and B). These results indicated that both BPA and MBP induced cytotoxicity through apoptosis induction in L2 cells.

### AMPK regulated endoplasmic reticulum (ER) stress signals in MBP-treated L2 alveolar epithelial cells

**Figure 7:**
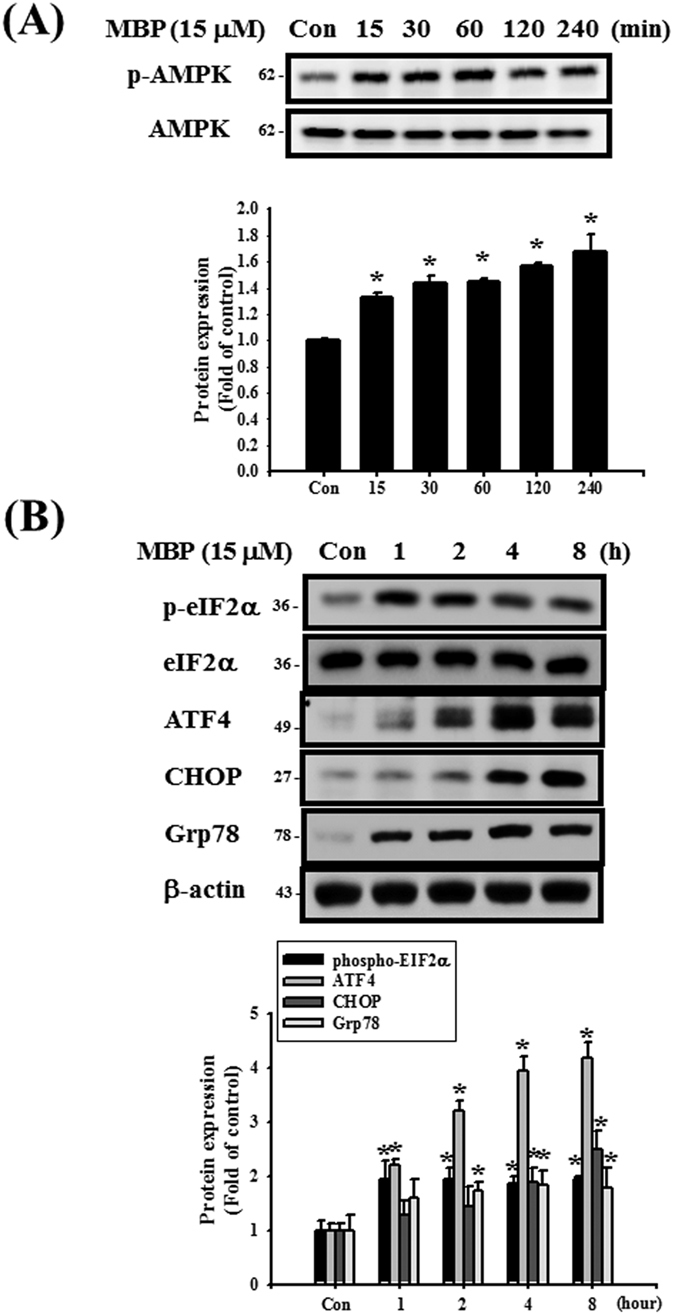
MBP induced AMPK phosphorylation and ER stress-related proteins expression in L2 alveolar epithelial cells. (**A**) Cells were treated with MBP (15 μM) for 15 to 240 min. The protein expressions of phospho-AMPK and AMPK were analyzed by Western blotting. (**B**) Cells were treated with MBP (15 μM) for 1–8 h. The protein expressions of ER stress-related proteins (phospho-eIF2α, eIF2α, ATF-4, CHOP, and Grp78) were analyzed by Western blotting. The protein expression of β-actin was as an internal control. The protein expressions were quantified by densitometry and analyzed by ImageQant TL 7.0 software. Data are presented as mean ± SEM of three independent experiments. *P < 0.05 versus vehicle control group. Con: control.

**Figure 8:**
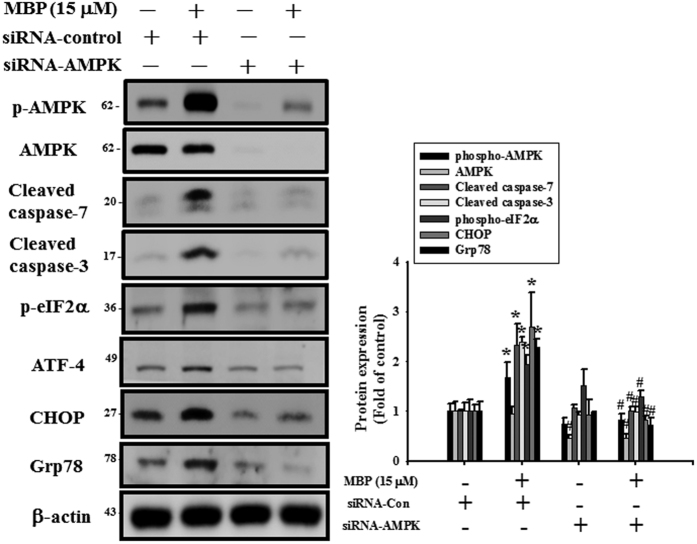
Involvement of AMPK in the MBP-induced apoptosis and ER stress-related signals in L2 alveolar epithelial cells. Cells were pretreated with siRNA-AMPK for 48 h, and then treated with MBP (15 μM) for 24 h. In (**A**) the protein levels of phospho-AMPK, AMPK, cleaved caspase-7, cleaved caspase-3, phospho-eIF2α, ATF-4, CHOP, and Grp78 were determined by Western blotting. The protein expression of β-actin was as an internal control. The protein expressions were quantified by densitometry and analyzed by ImageQant TL 7.0 software. Data are presented as mean ± SEM of three independent experiments. *P < 0.05 versus vehicle control group. ^#^*P* < 0.05 versus MBP combined with shRNA-control group.

**Figure 9:**
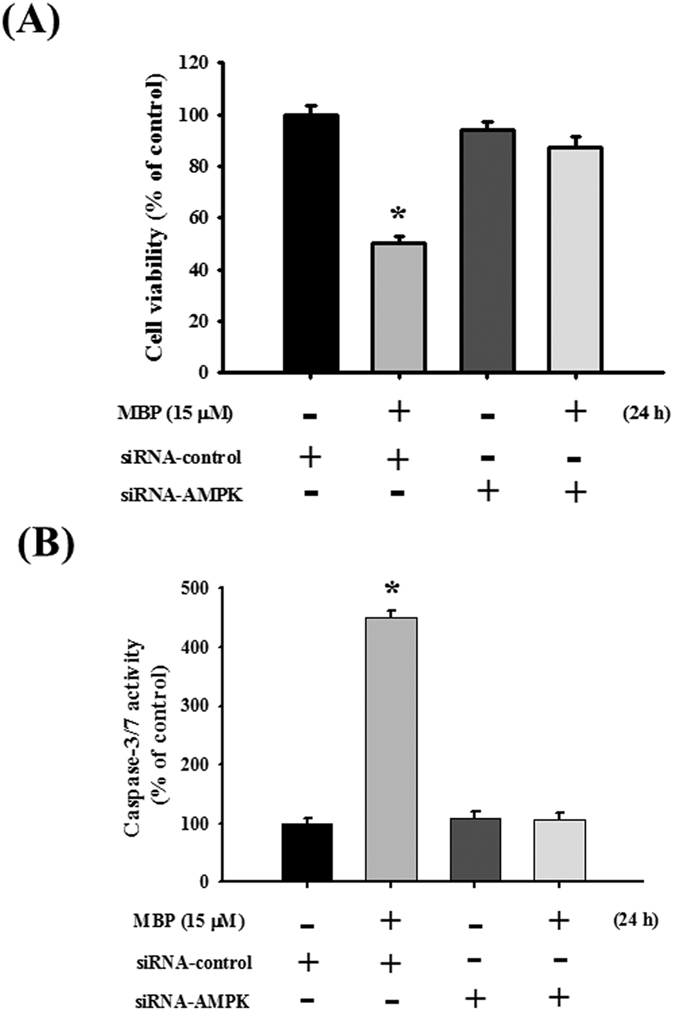
Involvement of AMPK in the MBP-induced cell viability reduction and caspase-3/7 activity induction in L2 alveolar epithelial cells. Cells were pretreated with siRNA-AMPK for 48 h, and then treated with MBP (15 μM) for 24 h. In (**A**) cell viability was determined by MTT assay. In (**B**) caspase-3/7 activity was determined by CaspACETM fluorometric activity assay. Data are presented as the mean ± SEM of three independent experiments. *P < 0.05 as compared with the vehicle control group.

As shown in Fig. 7A, the phosphorylation of AMPK was significantly increased after MBP treatment in L2 cells. The protein expressions of phospho-eIF2α, ATF4, CHOP, and Grp78 were also increased in MBP-treated L2 cells (Fig. 7B). For further investigate the role of AMPK in the induction of ER stress and apoptotic signals in MBP-treated L2 cells, the transfection of siRNA-AMPK was used. Results showed that siRNA-AMPK transfection reduced the protein expressions of phospho-AMPK, cleaved caspase-7, cleaved caspase-3, phospho-eIF2α, ATF-4, CHOP, and Grp78 in MBP-treated L2 cells (Fig. 8). Moreover, siRNA-AMPK transfection significantly reversed the decreased cell viability (Fig. 9A) and increased caspase-3/7 activity (Fig. 9B) in MBP-treated L2 cells. These results indicated that AMPK regulated MBP-induced ER-stress-related apoptotic signals in L2 cells.

On the other hand, MBP did not affect the mitochondrial transmembrane potential and protein expressions of Bcl-2 and Bax in L2 cells (Fig. S2-A and S2-B).

## Discussion

BPA exposure can occur through oral, dermal, and inhalation routes and metabolized to MBP^5^,^6^. BPA can be glucuronided by microsomal UGT2B1, which is an isoform of UDP-glucuronosyltransferase (UGT)^17^,^18^. It has been suggested that metabolic activation to MBP may occur in the detoxification pathway of BPA when glucuronidation cannot work efficiently, although the MBP metabolic activation may not be significant under usual circumstances^19^. Previous study has shown that the MBP metabolic activation can occur in the rat fetal livers^18^. In the late gestation of fetal liver, some CYP-dependent xenobiotic metabolisms have been considered to be working, such as CYP3A7, although the ability of CYP in fetal liver is limited than in adult liver^18^,^20^,^21^,^22^. A study has also found that MBP metabolic activation from BPA can occur under coexistence of rat liver microsomal and cytosolic fraction (S9 fraction) through CYP3A2 and CYP2C11^4^. Moreover, a study on the medaka (*Oryzias latipes*) suggested that MBP toxicity might be possible through its estrogenic activity^7^. MBP has a high affinity to His-524 of estrogen receptor (ER)-α and His-475 of ER-βthan BPA^23^. It has been suggested that MBP has about 250–1000 fold higher of estrogenic activity than BPA^7^,^18^,^23^. A study showed that 2 weeks of exposure to a dose of 10 mg/kg of BPA induced significant levels of DNA strand breaks in lung cells, but not in spleen cells, kidney cells, liver cells, or bone marrow lymphocytes^24^. In the lung, the alveolar epithelium is the first line of defense against microbes, toxicants and other foreign matter in the external environment^12^. Therefore, it may be supposed that MBP is an important active metabolite associated with pulmonary alveolar cell damage and lung dysfunction following BPA exposure. In the present study, the results of *in vitro* type 2 alveolar epithelial cell model and *ex vivo* isolated reperfused rat lung model showed that MBP is more potent than BPA in affecting alveolar cell viability, inducing apoptosis, and interfering with lung function.

The ER has functions in protein synthesis, including in the folding of secreted and membrane-bound proteins^25^. In type 2 alveolar cells, the ER is an important organelle that serves in surfactant protein synthesis and in the fusion of the lamellar body to the plasma membrane to permit secretion of the surfactant proteins^12^. Thus, ER stress induction might lead to a decrease in surfactant protein production and secretion in type 2 alveolar cells and cause lung dysfunction. Environmental cigarette smoke, bacterial infection, and allergens are known to disrupt ER homeostasis and induce ER stress-related apoptosis in lung epithelium^26^,^27^,^28^. ER stress-induced apoptosis can be initiated by the pancreatic ER kinase (PKR)-like ER kinase (PERK), the activation of transcription factor 6 (ATF6), or the expression of inositol-requiring enzymes1 (IRE1) pathway during unfolded protein response (UPR) signaling^25^. In ER stress, PERK, ATF6 and IRE1 activation follows the dissociation of Grp78 from these proteins. During PERK activation of ER stress, eukaryotic initiation factor 2α (eIF2α) is phosphorylated by PERK, inhibiting protein synthesis. The phosphorylation of eIF2α induces the cytoplasm-to-nuclear translocation of the transcription factor ATF4, which activates CHOP and other genes associated with the stress response. Another study also showed that up-regulation of Grp78 and induction of calpain II activated caspase-12 and caspase 3/7, leading to ER stress-induced cell death^29^. On the other hand, BPA exposure has been found to induce apoptosis in the Leydig and germ cells in the testes^30^. Asahi *et al*. have also shown that BPA induces apoptosis in mouse hepatocytes via an ER stress-associated pathway^31^. The effects of BPA or MBP on the Type 2 pulmonary alveolar epithelial cell apoptosis are still unclear. In the present study, we found that both BPA and MBP induced apoptosis in type 2 alveolar cells. MBP treatment induced apoptotic signals and ER stress, suggesting that an ER stress-regulated cell apoptosis signaling pathway is involved in the MBP-induced alveolar epithelial cell damage. On the other hand, mitochondrial pathway is also known to play an important role in triggering apoptosis^32^. However, our results indicated that MBP did not affect the mitochondrial transmembrane potential and protein expression of Bcl-2 and Bax in alveolar epithelial cells,

Mammalian AMPK has been reported to be a heterotrimeric complex, comprising a catalytic subunit (α) and two regulatory subunits (β and γ). Phosphorylation of Thr172 of the α-subunit is required for AMPK activation^33^,^34^. It has been shown that AMPK plays a key role in regulating intracellular homeostasis under physiological and pathological conditions, including during reactive oxygen species (ROS) balance, cellular proliferation, autophagy, apoptosis, mitochondrial function, and genotoxic response^31^. However, the role of AMPK activation in cellular apoptosis is controversial. For example, a study reported that the tumor suppressor gene *folliculin* (*FLCN*) was associated with alveolar epithelial function. Loss of FLCN induced alveolar epithelial dysfunction. In FLCN-null mice, impairment of AMPK activation increases the level of cleaved caspase-3, which leads to alveolar epithelial apoptosis^35^. Another study showed that AMPK activator AICAR induces apoptotic cell death and diminishes adiposity in adipocytes^36^. In the present study, MBP significantly induced AMPK phosphorylation in L2 cells. Transfection of siRNA-AMPK significantly inhibited AMPK phosphorylation and prevented ER stress and apoptosis induction and cell viability reduction in MBP-treated L2 cells. These results suggest that MBP induces type 2 alveolar cell damage through an AMPK-regulated cell apoptosis signaling pathway.

In conclusion, in this study, we demonstrate for the first time that BPA metabolite MBP induces pulmonary alveolar epithelial cell apoptosis and lung dysfunction determined by *in vitro* and *ex vivo* models. These findings suggest that MBP induces alveolar cell damage and lung dysfunction through an AMPK-regulated ER stress-related apoptosis signaling pathway.

## Materials and Methods

### Animals

Five-week-old male rats were used for isolated reperfused lung model. Animals were obtained from BioLASCO (Taipei, Taiwan). The protocols for animal studies were approved by the Institutional Animal Care and Use Committee of the China Medical University, Taichung, Taiwan, and the care and use of laboratory animals were conducted under the guidelines of the Animal Research Committee of the China Medical University. Animals were housed in a room at a constant temperature of 22 ± 2 °C with a 12 h light-dark cycle.

### Isolated reperfused lung model

The isolated perfused lung model has been shown to permit the real time collection and analysis of lung functions^37^,^38^. The experiments were carried out according to a modification of our previous method^39^. Animals were anesthetized (ketamine and xylazine) and the tracheae were intubated through a tracheostomy. The intubated tracheal tube was connected to a volume-controlled ventilator, and the animal was ventilated at a rate of 60 breath/min, a tidal volume of 10 mL/kg, and a positive end-expiratory pressure of 2 cmH_2_O with a gas mixture of 21% O_2_ and 5% CO_2_. Then, a median laparo-sternotomy was performed, the main pulmonary artery was cannulated through a right ventriculotomy, and the left atrium was cannulated via a left ventriculotomy. The cannulated catheters, with the flare at the tip, were sutured in place. The heart, lungs, and mediastinal structures were removed *en bloc* and suspended from a strain gauge force-displacement transducer (MLTF050/ST) (ADInstruments, Castle Hill, Australia) inside a humidified chamber to monitor weight changes. After a 30 min of steady-state period, the BPA (25, 50, and 75 μM) and MBP (5, 10, and 15 μM) were added to 100 mL of perfusate (a mixture of Krebs-Henseleit buffer with albumin), and continuously circulating in the lung for 120 min. The mean artery pressure (P_a_) and pulmonary capillary pressure (P_c_) were measured by pressure transducers (MLT0380/D) (ADInstruments).

### Measurement of pulmonary capillary filtration coefficient

After the lungs reached an isogravimetric state, the venous reservoir was rapidly elevated to increase P_v_ by 10 cm H_2_O. The increase in lung weight was recorded over time (△wt/△t). The initial 3-min period of weight gain represents vascular distension and recruitment and is not a reflection of capillary permeability. The △wt/△t between 4 and 10 min represents increased transvascular fluid flux secondary to increased capillary permeability. This later △wt/△t could be analyzed by linear regression of the log10-weight change per min. The initial rate of weight gain was calculated by extrapolation of △wt/△t to time zero^40^.

### Wet-to-dry weight ratio

At the windup of the experiment, all lungs were dissected free of non-pulmonary tissue and weighed and then dried to a constant weight at 60 °C. The wet-to-dry ratio was obtained by splitting up the wet weight by the final dried weight.

### Cell culture

Rat lung type 2 alveolar pneumonocytes-derived L2 cells were purchased from ATCC (CCL-149^TM^). Cells were cultured in RPMI-1640 media supplemented with 10% fetal bovine serum and 1% penicillin-streptomycin, under a 5% CO_2_ and 95% air mixture at 37 °C in a humid chamber.

### Cell viability assay

Cell viability was determined by 3-(4,5-dimethylthiazol- 2-yl)-2,5-diphenyltetrazolium bromide (MTT, Sigma) assay. L2 cells were counted and cultured in 24-well plates (5 × 10^4^ cells/well). After treatment of cells with or without tested materials for 24 h, cells were washed with PBS. MTT (0.2 mg/ml) was then added to each well and the mixture was incubated for 4 h at 37 °C. The media containing the indicated drugs was removed, and 1 mL dimethyl sulfoxide (DMSO) was added to dissolve blue formazan crystals that developed. The absorbance was measured at 570 nm using an enzyme-linked immunosorbent assay reader (Thermo Fisher Scientific, Waltham, MA, USA).

### Cell cycle analysis

Cells were trypsin-detached and washed with twice of PBS and then resuspended with 1 mL of ice-cold 70% (v/v) ethanol and stored at 4 °C for 24 h. Cells were washed with twice of PBS and stained with 50 μg/mL propidium iodide (PI) and 10 μg/mL ribonuclease (RNase) in PBS at 4 °C for 30 min in the dark environment. The cell cycle was analyzed by flow cytometry (FACScalibur, Becton Dickinson, Sunnyvale, CA, USA).

### Caspase-3/7 activity

An FLICA DEVD-FMK caspase 3/7 assay kit was used to analyze caspase3/7 activity (ImmunoChemistry Technologies, LLC, Bloomington, MN, USA). Briefly, the cells were counted and cultured in 24-well plates (5 × 10^4^ cells/well). After treatment of cells with or without tested materials for 24 h, cells were next collected into 1.5 mL tubes and centrifuged at 200 × *g* for 5 min at 4 °C. Cells were then washed twice with PBS and stained with fluorescent probes for 10 min in a dark environment at room temperature. A flow cytometer was used to analyze fluorescence intensity (FACScalibur, Becton Dickinson, Sunnyvale, CA, USA).

### Annexin-V-FITC assay

An annexin-V-FITC apoptosis detection kit (BioVision, CA, USA) was used to analyze apoptosis event. Cells were counted and cultured in 24-well plates (5 × 10^4^ cells/well) and treated with the indicated drugs for 24 h. The cells were then detached and washed twice with PBS, after which cells were stained with annexin-V-FITC for 20 min at room temperature. Finally, the cells were washed twice with PBS, and fluorescence was detected by flow cytometry (FACScalibur, Becton Dickinson, Sunnyvale, CA, USA).

### Small interfering RNA (siRNA) transfection

An AMPK-directed siRNA and a scrambled siRNA were purchased from Santa Cruz Biotechnologies (Santa Cruz, CA, USA). Briefly, cells were counted and cultured in 24-well plates (5 × 10^4^ cells/well). After overnight incubation, cells were washed twice with PBS and cultured in Opti-MEM medium for 4 h. Transfections of siRNA-AMPK and siRNA-scrambled were performed using Lipofectamine 2000 (Invitrogen, Life Technologies, Inc., USA) for 48 h.

### Western blot analysis

Cells were washed twice with PBS and lysed. The lysate samples were then centrifuged at 14000 × *rpm* for 20 min at 4 °C. Supernatants were collected, and equal amounts of protein (50 μg per lane) were subjected to electrophoresis on 10% (W/V) SDS-polyacrylamide gels and then transferred to polyvinylidenedifluoride (PVDF) membranes. The membranes were blocked with 5% nonfat dry milk in PBST (PBS and 0.05% Tween 20) for 1 h, washed twice with 0.1% PBST, and incubated with primary antibodies for ATF-4, CHOP, pro-caspase-7, cleaved caspase-7 (Cell Signaling Technologies, MA, USA), Grp-78, pro-caspase-3, cleaved caspase-3, Bcl-2, Bax (Santa Cruz, CA, USA), phospho-AMPK, AMPK, phospho-eukaryotic initiation factor 2α (eIF2α), and eIF-2α (Abcam, Cambridge, MA, USA) for 1 h. The membranes were then washed with 0.1% PBST and incubated with secondary antibodies that were conjugated to horseradish peroxidase for 45 min. Antibody-reactive bands were revealed using enhanced chemiluminescence reagents (Amersham Biosciences, Sweden) and exposed to Fuji radiographic film. The protein expression of β-actin was as an internal control. The protein expressions were quantified by densitometry and analyzed by ImageQant TL 7.0 software.

### Statistical analysis

The data are presented as the mean ± SEM. One-way ANOVA was used for the analysis of multiple groups. Duncan’s post hoc test was applied to identify group differences. *P* values less than 0.05 were considered to be significant. The statistical package SPSS 11.0 for Windows (SPSS Inc., Chicago, IL, USA) was used for all statistical analyses.

## Additional Information

**How to cite this article**: Liu, S.-H. *et al*. Effects of Bisphenol A Metabolite 4-Methyl-2,4-bis(4-hydroxyphenyl)pent-1-ene on Lung Function and Type 2 Pulmonary Alveolar Epithelial Cell Growth. *Sci. Rep.* **6**, 39254; doi: 10.1038/srep39254 (2016).

**Publisher's note:** Springer Nature remains neutral with regard to jurisdictional claims in published maps and institutional affiliations.

## Supplementary Material

Supplementary Figures

## References

[b1] Takayanagi S. . Endocrine disruptor bisphenol A strongly binds to human estrogen-related receptor-γ (ERRγ) with high constitutive activity. Toxicol Lett 167, 95–105 (2006). 17049190 10.1016/j.toxlet.2006.08.012

[b2] Singh S. & Li S. S. Epigenetic effects of environmental chemicals bisphenol A and phthalates. Int J Mol Sci 13, 10143–10153 (2012). 22949852 10.3390/ijms130810143 PMC3431850

[b3] Van Winkle L. S., Murphy S. R., Boetticher M. V. & VandeVoort C. A. Fetal exposure of rhesus macaques to bisphenol a alters cellular development of the conducting airway by changing epithelial secretory product expression. Environ Health Perspect 121, 912–918 (2013). 23757601 10.1289/ehp.1206064 PMC3734491

[b4] Yoshihara S. . Potent estrogenic metabolites of bisphenol A and bisphenol B formed by rat liver S9 fraction: their structures and estrogenic potency. Toxicol Sci 78, 50–59 (2004). 14691209 10.1093/toxsci/kfh047

[b5] Lim D. S. . Potential risk of bisphenol A migration from polycarbonate containers after heating, boiling, and microwaving. J Toxicol Environ Health A 72, 1285–1291 (2009). 20077198 10.1080/15287390903212329

[b6] Donohue K. M. . Prenatal and postnatal bisphenol A exposure and asthma development among inner-city children. J Allergy Clin Immunol 131, 736–742 (2013). 23452902 10.1016/j.jaci.2012.12.1573 PMC3643970

[b7] Ishibashi H. . Toxicity to early life stages and an estrogenic effect of a bisphenol A metabolite, 4-methyl-2,4-bis(4-hydroxyphenyl)pent-1-ene on the medaka (Oryzias latipes). Life Sci 77, 2643–2655 (2005). 15961118 10.1016/j.lfs.2005.03.025

[b8] Midoro-Horiuti T., Tiwari R., Watson C. S. & Goldblum R. M. Maternal bisphenol a exposure promotes the development of experimental asthma in mouse pups. Environ Health Perspect 118, 273–277 (2010). 20123615 10.1289/ehp.0901259 PMC2831929

[b9] Spanier A. J. . Bisphenol A exposure and the development of wheeze and lung function in children through age 5 years. JAMA Pediatr 168, 1131–1137 (2014). 25286153 10.1001/jamapediatrics.2014.1397 PMC4535321

[b10] Lee M. H. . Enhanced interleukin-4 production in CD4+ T cells and elevated immunoglobulin E levels in antigen-primed mice by bisphenol A and nonylphenol, endocrine disruptors: involvement of nuclear factor-AT and Ca2+. Immunology 109, 76–86 (2003). 12709020 10.1046/j.1365-2567.2003.01631.x PMC1782943

[b11] Miller B. E. & Hook G. E. Hypertrophy and hyperplasia of alveolar type II cells in response to silica and other pulmonary toxicants. Environ Health Perspect 85, 15–23 (1990). 2166657 10.1289/ehp.85-1568321 PMC1568321

[b12] Aoshiba K. & Nagai A. Oxidative stress, cell death, and other damage to alveolar epithelial cells induced by cigarette smoke. Tob Induc Dis 1, 219–226 (2003). 19570263 10.1186/1617-9625-1-3-219 PMC2671551

[b13] Hohlfeld J., Fabel H. & Hamm H. The role of pulmonary surfactant in obstructive airways disease. Eur Respir J 10, 482–491 (1997). 9042653 10.1183/09031936.97.10020482

[b14] Guo X. . Surfactant protein gene A, B, and D marker alleles in chronic obstructive pulmonary disease of a Mexican population. Eur Respir J 18, 482–490 (2001). 11589345 10.1183/09031936.01.00043401

[b15] Vlachaki E. M. . Altered surfactant protein-A expression in type II pneumocytes in COPD. Chest 137, 37–45 (2010). 19741063 10.1378/chest.09-1029

[b16] Yang W. . Alveolar type II epithelial cell dysfunction in rat experimental hepatopulmonary syndrome (HPS). PLoS One 9, e113451 (2014). 25419825 10.1371/journal.pone.0113451 PMC4242631

[b17] Yokota H. . Glucuronidation of the environmental oestro-gen bisphenol A by an isoform of UDP-glucuronosyltransferase, UGT2B1, in the rat liver. Biochem J 340, 405–409 (1999). 10333482 PMC1220264

[b18] Okuda K., Takiguchi M. & Yoshihara S. *In vivo* estrogenic potential of 4-methyl-2,4-bis(4-hydroxyphenyl)pent-1-ene, an active metabolite of bisphenol A, in uterus of ovariectomized rat. Toxicol Lett 197, 7–11 (2010). 20435109 10.1016/j.toxlet.2010.04.017

[b19] Okuda K., Fukuuchi T., Takiguchi M. & Yoshihara S. Novel pathway of metabolic activation of bisphenol A-related compounds for estrogenic activity. Drug Metab Dispos 39, 1696–1703 (2011). 21636669 10.1124/dmd.111.040121

[b20] Cresteil T. . Cytochrome P-450 isoenzyme content and monooxygenase activities in rat liver: effect of ontogenesis and pretreatment by phenobarbital and 3-methylcholanthrene. J Pharmacol Exp Ther 236, 269–276 (1986). 3941398

[b21] Kitada M. . P-450 HFLa, a form of cytochrome P-450 purified from human fetal livers, is the 16 alpha-hydroxylase of dehydroepiandrosterone 3-sulfate. J Biol Chem 262, 13534–13537 (1987). 3654629

[b22] Raucy J. L. & Carpenter S. J. The expression of xenobiotic-metabolizing cytochromes P450 in fetal tissues. J Pharmacol Toxicol Methods 29, 121–128 (1993). 8364226 10.1016/1056-8719(93)90062-j

[b23] Baker M. E. & Chandsawangbhuwana C. 3D models of MBP, a biologically active metabolite of bisphenol A, in human estrogen receptor alpha and estrogen receptor beta. PLoS One 7, e46078 (2012). 23056236 10.1371/journal.pone.0046078 PMC3464279

[b24] Gajowik A., Radzikowska J. & Dobrzynska M. M. Genotoxic effects of bisphenol A on somatic cells of female mice, alone and in combination with X-rays. Mutat Res 757, 120–124 (2013). 23954285 10.1016/j.mrgentox.2013.07.006

[b25] Szegezdi E., Logue S. E., Gorman A. M. & Samali A. Mediators of endoplasmic reticulum stress-induced apoptosis. EMBO Rep 7, 880–885 (2006). 16953201 10.1038/sj.embor.7400779 PMC1559676

[b26] Hengstermann A. & Muller T. Endoplasmic reticulum stress induced by aqueous extracts of cigarette smoke in 3T3 cells activates the unfolded-protein-response-dependent PERK pathway of cell survival. Free Radic Biol Med 44, 1097–1107 (2008). 18206657 10.1016/j.freeradbiomed.2007.12.009

[b27] Ribeiro C. M. & Boucher R. C. Role of endoplasmic reticulum stress in cystic fibrosis-related airway inflammatory responses. Proc Am Thorac Soc 7, 387–394 (2010). 21030518 10.1513/pats.201001-017AW PMC3136959

[b28] Schroeder B. W. . AGR2 is induced in asthma and promotes allergen-induced mucin overproduction. Am J Respir Cell Mol Biol 47, 178–185 (2012). 22403803 10.1165/rcmb.2011-0421OC PMC3423459

[b29] Martinez J. A. . Calpain and caspase processing of caspase-12 contribute to the ER stress-induced cell death pathway in differentiated PC12 cells. Apoptosis 15, 1480–1493 (2010). 20640600 10.1007/s10495-010-0526-4

[b30] Li Y. J. . Bisphenol A exposure induces apoptosis and upregulation of Fas/FasL and caspase-3 expression in the testes of mice. Toxicol Sci 108, 427–436 (2009). 19193734 10.1093/toxsci/kfp024

[b31] Asahi J. . Bisphenol A induces endoplasmic reticulum stress-associated apoptosis in mouse non-parenchymal hepatocytes. Life Sci 87, 431–438 (2010). 20807545 10.1016/j.lfs.2010.08.007

[b32] Chen Y. W., Yang Y. T., Hung D. Z., Su C. C. & Chen K. L. Paraquat induced lung alveolar epithelial cell apoptosis via Nrf-2-regulated mitochondrial dysfunction and ER stress. Arch Toxicol 86, 1547–1548 (2012). 22678742 10.1007/s00204-012-0873-8

[b33] Kadappu K. K. . Changes in left atrial volume in diabetes mellitus: more than diastolic dysfunction? Eur Heart J Cardiovasc Imaging 13, 1016–1023 (2012). 22544873 10.1093/ehjci/jes084

[b34] Wang S., Song P. & Zou M. H. AMP-activated protein kinase, stress responses and cardiovascular diseases. Clin Sci (Lond) 122, 555–573 (2012). 22390198 10.1042/CS20110625 PMC3367961

[b35] Goncharova E. A. . Folliculin controls lung alveolar enlargement and epithelial cell survival through E-cadherin, LKB1, and AMPK. Cell Rep 7, 412–423 (2014). 24726356 10.1016/j.celrep.2014.03.025 PMC4034569

[b36] Dagon Y., Avraham Y. & Berry E. M. AMPK activation regulates apoptosis, adipogenesis, and lipolysis by eIF2alpha in adipocytes. Biochem Biophys Res Commun 340, 43–47 (2006). 16377306 10.1016/j.bbrc.2005.11.159

[b37] Niemeier R. W. The isolated perfused lung. Environ Health Perspect 56, 35–41 (1984). 6383800 10.1289/ehp.845635 PMC1568207

[b38] Bernard C. E., Dahlby R. & Hoener B. An isolated perfused lung model with real time data collection and analysis of lung function. J Pharmacol Toxicol Methods 38, 41–46 (1997). 9339415 10.1016/s1056-8719(97)00047-6

[b39] Lai Y. L., Chu S. J., Ma M. C. & Chen C. F. Temporal increase in the reactivity of pulmonary vasculature to substance P in chronically hypoxic rats. Am J Physiol Regul Integr Comp Physiol 282, R858–864 (2002). 11832408 10.1152/ajpregu.00429.2001

[b40] Drake R., Gaar K. A. & Taylor A. E. Estimation of the filtration coefficient of pulmonary exchange vessels. Am J Physiol 234, H266–274 (1978). 629362 10.1152/ajpheart.1978.234.3.H266

