# Supplementary material for: Effects of Bisphenol A Metabolite 4-Methyl-2,4-bis(4-hydroxyphenyl)pent-1-ene on Lung Function and Type 2 Pulmonary Alveolar Epithelial Cell Growth
Source: Sci Rep. 2016 Dec 16;6:39254. doi: 10.1038/srep39254 (PMC5159875; doi:10.1038/srep39254)
Supplement: Supplementary Figures [file srep39254-s1.pdf]

SUPPLEMENTARY INFORMATION

Effects of Bisphenol A Metabolite 4-Methyl-2,4-bis(4-hydroxyphenyl)pent-1-ene on Lung Function and Type 2 Pulmonary Alveolar Epithelial Cell Growth

Shing-Hwa Liu,[1,2,]* Chin-Chuan Su,[3] Kuan-I Lee,[4,]* Ya-Wen Chen[5]

[1]Institute of Toxicology, College of Medicine, National Taiwan University, Taipei, Taiwan; [2]Department of Medical Research, China Medical University Hospital, China Medical University, Taichung, Taiwan; [3]Department of Otorhinolaryngology, Head and Neck Surgery, Changhua Christian Hospital, Changhua County, Taiwan; [4]Department of Emergency, Taichung Tzuchi Hospital, Taichung, Taiwan; [5]Department of Physiology and Graduate Institute of Basic Medical Science, College of Medicine, China Medical University, Taichung, Taiwan

*These authors contributed equally to this study.

Address correspondence to Ya-Wen Chen, Department of Physiology and Graduate Institute of Basic Medical Science, College of Medicine, China Medical University, Taichung, Taiwan. E-mail: ywc@mail.cmu.edu.tw; Tel.: +886 4 22053366 ext. 2192.

Supplementary Figures

(A)

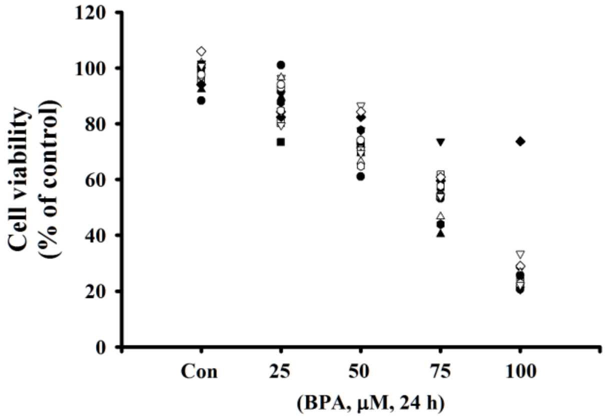

(B)

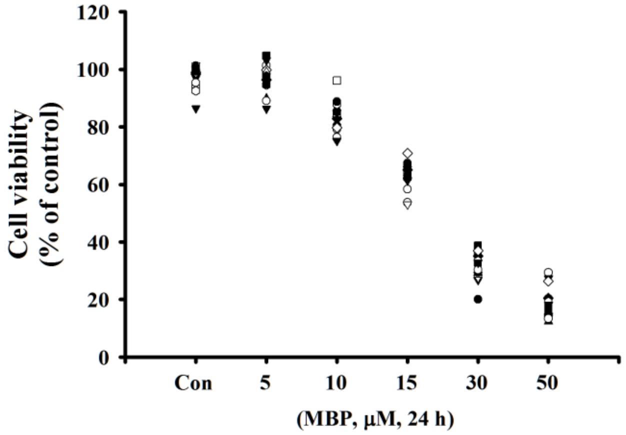

Figure S1. BPA and its metabolite MBP suppressed cell viability in L2 alveolar epithelial cells. Cells were treated with BPA (25-100 μM, A) or MBP (5-50 μM, B) for 24 h. Cell viability was determined by MTT assay. Data are presented as mean ± SEM of three independent experiments. Con: control.

(A)

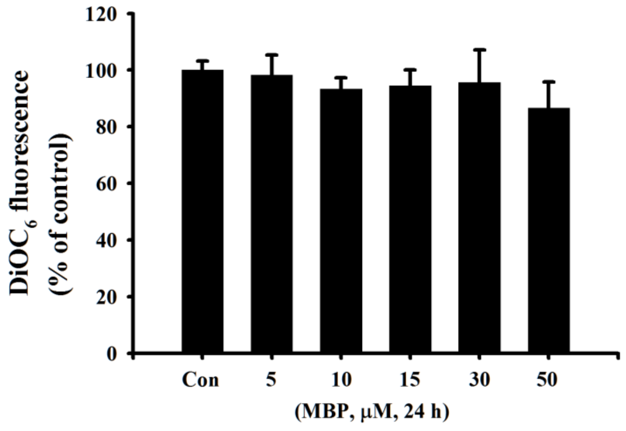

(B)

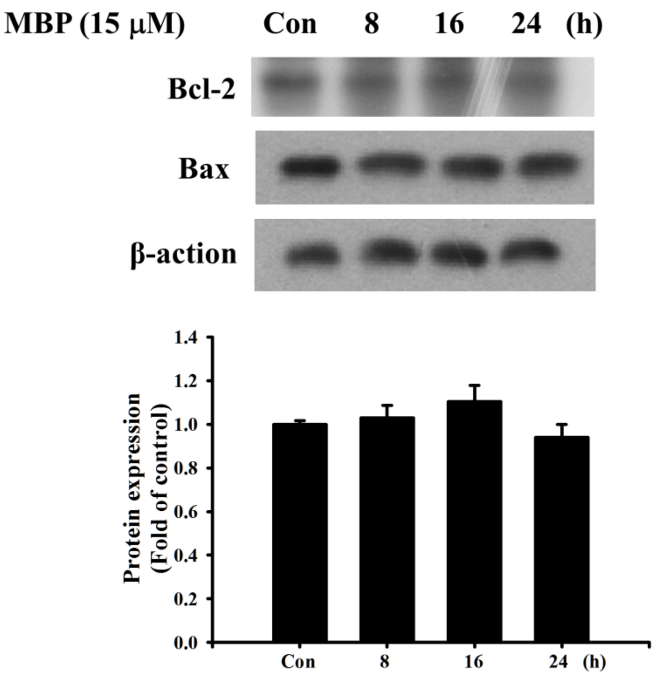

Figure S2. Effects of MBP on mitochondrial transmembrane potential (MMP) and protein expressions of Bcl-2 and Bax in L2 alveolar epithelial cells. (A) Cells were treated MBP (5 to 50 μM) for 24 h. The MMP were analyzed by DiOC6 staining with flowcytometry. Data are presented as mean ± SEM of three independent experiments. (B) Cells were treated MBP (15 μM) for 24 h. The protein expressions of Bcl-2 and Bax were analyzed by Western blot analysis. Data are presented as mean ± SEM of three independent experiments.

Supplementary Methods

Mitochondrial transmembrane potential (MMP) assay

The flowcytometery was used to detect the MMP in cells. After cells were treated with MBP (5 to 50 μM) or vehicle, cells were washing twice of PBS and treated with $DiOC_6$ for 30 min. And then, the MMP was analysis by flowcytometer (FACScalibur, Becton Dickinson, Sunnyvale, CA, USA).

Western blot analysis

Cells were washed twice with PBS and lysed. The lysate samples were then centrifuged at 14000 x rpm for 20 min at 4^oC. Supernatants were collected, and equal amounts of protein (50 μg per lane) were subjected to electrophoresis on 10% (W/V) SDS-polyacrylamide gels and then transferred to polyvinylidenedifluoride (PVDF) membranes. The membranes were blocked with 5% nonfat dry milk in PBST (PBS and 0.05% Tween 20) for 1 h, washed twice with 0.1% PBST, and incubated with primary antibodies for Bcl-2, Bax, and β-actin (Santa Cruz, CA, USA) for 1 h. The membranes were then washed with 0.1% PBST and incubated with secondary antibodies that were conjugated to horseradish peroxidase for 45 min. Antibody-reactive bands were revealed using enhanced chemiluminescence reagents (Amersham Biosciences, Sweden) and exposed to Fuji radiographic film. The protein expression of β-actin was as an internal control. The protein expressions were quantified by densitometry and analyzed by ImageQant TL 7.0 software.
